# Meningococcus serogroup C clonal complex ST-10217 outbreak in Zamfara State, Northern Nigeria

**DOI:** 10.1038/s41598-018-32475-2

**Published:** 2018-09-21

**Authors:** Brenda A. Kwambana-Adams, Rahab C. Amaza, Catherine Okoi, Murtala Rabiu, Archibald Worwui, Ebenezer Foster-Nyarko, Bernard Ebruke, Abdul K. Sesay, Madikay Senghore, Abdullahi S. Umar, Rabi Usman, Adamu Atiku, Garba Abdullahi, Yahaya Buhari, Rabiu Sani, Husaini U. Bako, Bashir Abdullahi, Alliyu I. Yarima, Badaru Sikiru, Aderinola Olaolu Moses, Michael O. Popoola, Eme Ekeng, Adebola Olayinka, Nwando Mba, Adamu Kankia, Ibrahim N. Mamadu, Ifeanyi Okudo, Mary Stephen, Olivier Ronveaux, Jason Busuttil, Jason M. Mwenda, Mohammed Abdulaziz, Sulaiman A. Gummi, Adebayo Adedeji, Andre Bita, Linda Omar, Mamoudou Harouna Djingarey, Wondimagegnehu Alemu, Umberto D’Alessandro, Chikwe Ihekweazu, Martin Antonio

**Affiliations:** 1 World Health Organization, Collaborating Centre for New Vaccines Surveillance, Medical Research Council Unit The Gambia at London School of Hygiene & Tropical Medicine, Atlantic Boulevard, Fajara, PO Box 273, Banjul, The Gambia; 2 Nigeria Center for Disease Control, Abuja, Nigeria; 3 Ahmad Sani Yariman Bakura Specialist Hospital Gusau, Zamfara State, Gusau, Nigeria; 4 Zamfara State Ministry of Health, Gusau, Nigeria; 5 Africa Centres for Diseases Control and Prevention, Addis Ababa, Ethiopia; 6 World Health Organization, Country Office Nigeria, Abuja, Nigeria; 7 World Health Organization, Geneva, Switzerland; 8 UK-Public Health Rapid Support Team, Public Health England, Salisbury, UK; 9 World Health Organization, Regional office for Africa, Brazzaville, Congo; 10 World Health Organization Inter-Country Support Teams for West Africa, Ouagadougou, Burkina Faso; 11 Disease Control and Elimination Theme, Medical Research Council Unit The Gambia at London School of Hygiene & Tropical Medicine, Atlantic Boulevard, Fajara, PO Box 273, Banjul, The Gambia; 12 Division of Microbiology & Immunity, Warwick Medical School, University of Warwick, Coventry, UK

## Abstract

After the successful roll out of MenAfriVac, Nigeria has experienced sequential meningitis outbreaks attributed to meningococcus serogroup C (NmC). Zamfara State in North-western Nigeria recently was at the epicentre of the largest NmC outbreak in the 21^st^ Century with 7,140 suspected meningitis cases and 553 deaths reported between December 2016 and May 2017. The overall attack rate was 155 per 100,000 population and children 5–14 years accounted for 47% (3,369/7,140) of suspected cases. The case fatality rate (CFR) among children 5–9 years was 10%, double that reported among adults ≥ 30 years (5%). NmC and pneumococcus accounted for 94% (172/184) and 5% (9/184) of the laboratory-confirmed cases, respectively. The sequenced NmC belonged to the ST-10217 clonal complex (CC). All serotyped pneumococci were PCV10 serotypes. The emergence of NmC ST-10217 CC outbreaks threatens the public health gains made by MenAfriVac, which calls for an urgent strategic action against meningitis outbreaks.

## Introduction

The “meningitis belt” spans twenty-six contiguous countries across Africa and is characterized by large recurrent meningitis epidemics and frequent seasonal outbreaks^1^. Within the “meningitis belt”, *Neisseria meningitidis* (meningococcus) accounts for almost all recurrent epidemics. The entire northern region of sub-Saharan Africa’s most populous country, Nigeria, falls within the meningitis belt. Nigeria has a long history of large-scale meningitis outbreaks dating as far as the early 1900s; in 1921, an outbreak in northern Nigeria caused 46,000 deaths^2^. The most recent largest meningitis outbreak occurred in 1996, with over 80,000 suspected cases^3,4^.

The bulk of epidemic meningococcal disease outbreaks in Nigeria and across the “meningitis belt” were previously attributed to meningococcus serogroup A (NmA)^5^. MenAfriVac is a conjugate vaccine that is very immunogenic and, provides effective protection against NmA^6^. MenAfriVac campaigns have prevented NmA epidemics and reduced the incidence of suspected meningitis by nearly two-thirds^7^. In 2011, nearly 16 million people in five high priority states (Zamfara, Katsina, Jigawa Bauchi and Gombe) in northern Nigeria received MenAfriVac^8^.

In 1975, Nigeria experienced a large NmC epidemic associated with relatively high rates of meningococcaemia and high case fatality rates as seen in the recent outbreaks^2,9^. Before 2013, the latest outbreak of NmC in Africa had occurred in 1979 in Burkina Faso^10^. Sequential outbreaks of meningococcus serogroup C (NmC) occurred in Nigeria in 2013 and 2014 in Nigeria^11,12^ despite the roll out of MenAfriVac campaigns. These outbreaks were followed by a larger outbreak in Niger in 2015^13^. All the recent outbreaks were attributed to a novel strain of NmC sequence type (ST)-10217 whose emergence threatens the public health gains made by the MenAfriVac^14–16^.

Monovalent (C) and quadrivalent (A,C,W,Y) formulations of the meningococcal conjugate vaccine are available and The World Health Organization (WHO) recommends that countries with endemic or frequent epidemics of meningococcal disease should implement large-scale vaccination campaigns^17,18^. However, the rollout of vaccines that protect against NmC has been slow and tends to be reactive during outbreaks. The major constraints to preventive vaccination campaigns include high vaccine cost, limited vaccine availability and competing public health interests^15^. During epidemics, reactive vaccination campaigns are further hampered by low lumbar puncture rates among suspected cases and inadequate laboratory characterisation of causative pathogens that should guide response^19^.

Between December 2016 and May 2017, a total of 14,280 suspected meningitis cases were reported across 23 of the 36 states in Nigeria. The descriptive characterization of the 2016–2017 cerebrospinal meningitis outbreak in Nigeria is reported elsewhere^20^. There were 1,145 deaths (8% case fatality rate) amongst suspected cases. Zamfara and Sokoto states were at the epicenter of the meningitis epidemic in Nigeria. The World Health Organization Collaborating Center (WHO CC) for New Vaccines hosted at the Medical Research Council Unit The Gambia at London School of Hygiene & Tropical Medicine, supports surveillance of invasive bacterial diseases (IBD) in West and Central Africa. The WHO CC with its partners, supported the Nigeria Center for Disease Control (NCDC) to confirm the causative agent of the outbreak in Zamfara state. The WHO CC also trained and supported the laboratory teams at regional and district hospitals in Zamfara for bacteriologic analysis and processing of CSF specimens for molecular analysis.

## Results

A total of 7,140 suspected meningitis cases were reported in Zamfara State between 12^th^ December 2016 and 31^st^ May 2017. The attack rate was 155 per 100,000 population. A total of 553 deaths were reported among suspected cases and the case fatality ratio (CFR) was 8% in Zamfara state.

**Figure 1:**
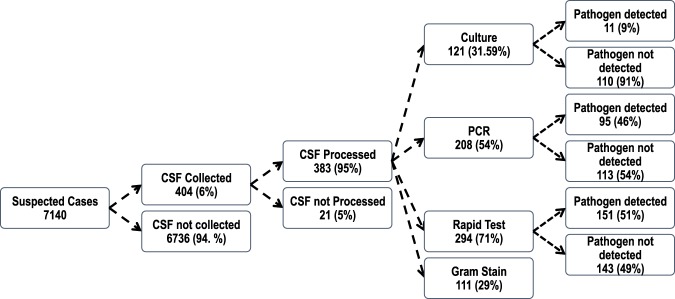
Summary of CSF specimen collection and processing in Zamfara State.

CSF specimens were collected from 404 (6%) suspected meningitis cases and almost all, 383 (95%), had at least one or all laboratory tests performed including bacteriologic culture, Gram stain, Rapid Test (Pastorex) and qPCR. The laboratory processing of CSF specimens is shown in Fig. 1. A pathogen was detected in 184 (48%) of the CSF specimens tested by PCR, rapid test and culture. Meningococcus was the dominant pathogen accounting for 173 (94%) of the confirmed cases. The characteristics of the suspected and confirmed meningitis cases are summarised in Table 1. Of the 112 CSF specimens tested by both PCR and Rapid Test, the concordance was 86 (77%). PCR detected an additional 19 Nm meningitis cases in addition to the 22 cases detected by both methods and 3 cases detected by Rapid Test only.

**Table 1:** Baseline characteristics of patients with suspected and confirmed meningitis in Zamfara State.

Characteristic	Category	Suspected Cases n (%)	Confirmed Cases n (%)
Gender	Female	3,566 (49.9%)	79 (42.7%)
	Male	3,574 (50.1%)	106 (57.3%)
Age	<1	37 (0.5%)	3 (6.5%)
	1–4	838 (11.7%)	17 (9.2%)
	5–9	1,640 (23.0%)	52 (28.1%)
	10–14	1,729 (24.2%)	58 (31.3%)
	15–29	2,089 (29.3%)	42 (22.7%)
	> = 30	803(11.3%)	12 (6.5%)
	Unknown	4 (0.05%)	1 (0.5%)
On set month	December	17 (0.2%)	0 (0.0%)
	January	180 (2.5%)	9 (4.9%)
	February	340 (4.8%)	38 (20.5%)
	March	2,174 (30.4%)	27 (14.6%)
	April	4,031 (56.5%)	51 (27.6%)
	May	353 (4.9%)	33 (17.8%)
	Unknown	45 (0.6%)	27 (14.6%)
Outcome	Alive	6,553 (91.8%)	156 (84.2%)
	Dead	553 (7.7%)	6 (3.2%)
	Unknown	34 (0.5%)	23 (12.4%)

NmC accounted for 172 (99%) of the meningococci detected. There was one case of meningococcus serogroup W (NmW) meningitis detected by latex rapid test. MLST performed directly from two CSF specimens that tested positive for NmC showed that they belonged to the ST-10217 CC. Unfortunately, the meningococcal isolates cultured from CSF specimens during the outbreak were lost due to the lack of appropriate storage conditions in Zamfara state. It was not possible to perform MLST on more NmC positive CSF specimens due to the limited availability of funds.

*Streptococcus pneumoniae*, the pneumococcus, accounted for 9 (5%) cases. All five of the serotyped pneumococci were PCV10 vaccine strains; three were serotype 19 F strains, one serotype 1 and one serotype 5. There were also single cases of confirmed Group B *Streptococcus* (GBS) and *H. influenzae*.

### Spatial and temporal characteristics of the outbreak

**Figure 2:**
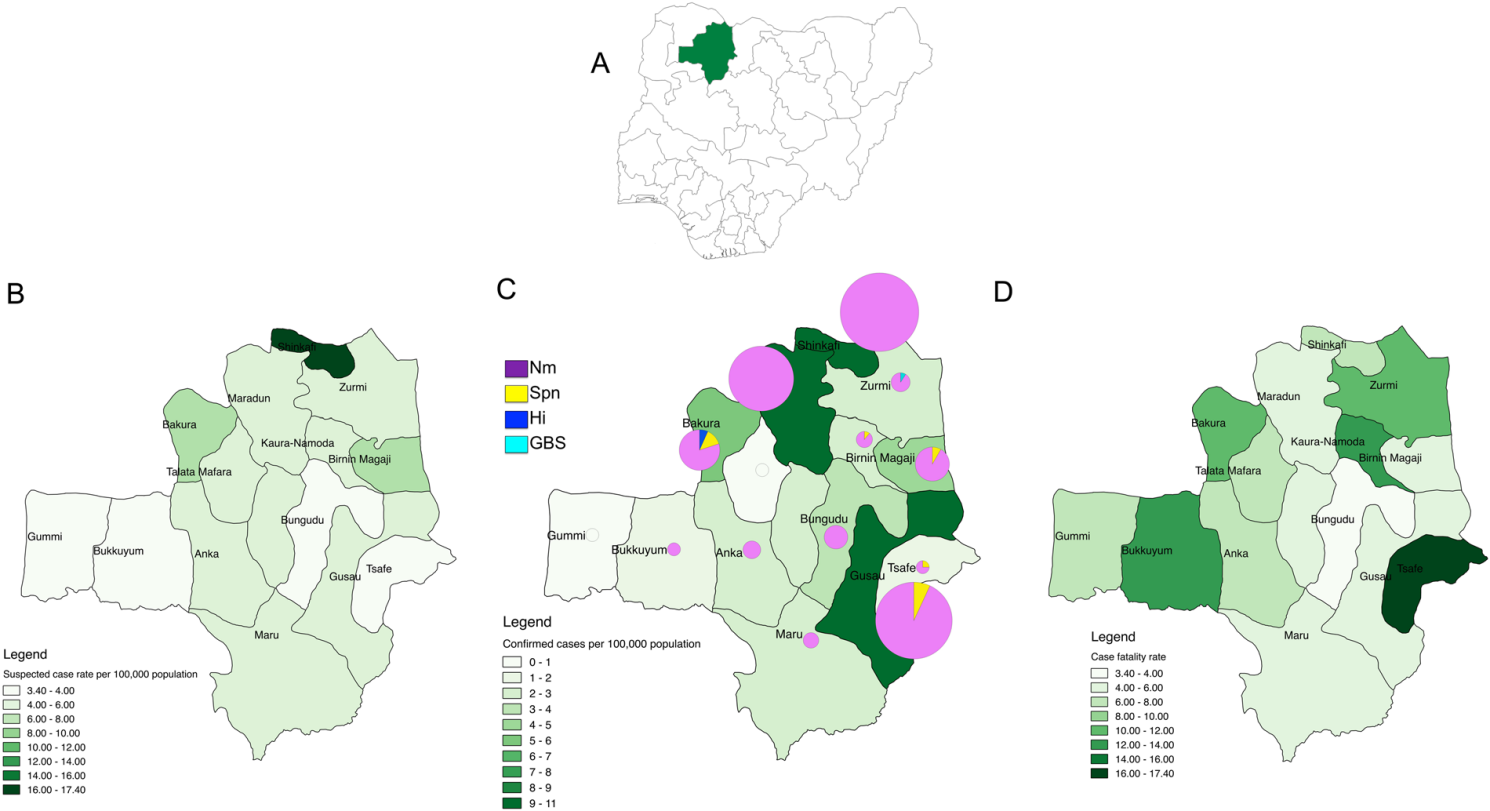
The geographical distribution of the meningitis outbreak in Zamfara State. The location of Zamfara state in Nigeria is shown in (**A**). Suspected cases per 100,000 population (**B**), laboratory-confirmed cases per 100,000 population (**C**) and case fatality rates (**D**) across LGAs are shown by colour gradient. The pie charts represent the pathogens detected in confirmed cases (**C**).

The epicentre of the outbreak in Zamfara state (Fig. 2A) was Shinkafi Local Government Area (LGA), which had an attack rate of 669 per 100,000 population and bore a fifth of all the suspected cases (Fig. 2B). Other LGAs with high attack rates were Bakura and Birnin Magaji, which had 210 and 255 suspected cases per 100,000 population, respectively. Gusau, the state capital, accounted for 12% of the suspected cases reported, although many of these suspected cases may have been referrals from other LGAs (Fig. 2B). Shinkafi, Maradun and Gusau had the highest confirmed cases, over 8 per 100,000 population. Meningococcus was the dominant pathogen across all the LGAs in Zamfara state. Pneumococcal meningitis was reported in Bakura, Gusau, Magaji and Bimiri (Fig. 2C).

Although Tsafe had a relatively low attack rate of 44.4 per 100,000, this LGA had the highest CFR of 17% (Fig. 2D). Other LGAs with high CFRs were Kaura Namoda (12%), Bukkuyum (13%) and Bakura (11%). Suspected meningitis cases from Tsafe LGA were more than three times likely to die compared to suspected meningitis cases from Gusau (p < 0.01) (Table 2). Likewise, suspected meningitis cases from Bakura and Kaura Namoda LGAs had significantly higher odds of dying than suspected meningitis cases from Gusau LGA (Table 2). Suspected meningitis cases from Maradun and Bungudu had CFRs less than 5% (Fig. 2D) and were significantly less likely to die than suspected from the state capital, Gusau.

**Table 2:** Factors associated with mortality among suspected meningitis cases in Zamfara state.

	Category	Suspected Cases	Deaths (%)	OR	95%CI	P-value
Age group (years)	≥30	802	39 (4.9)	1.00		
	<1	37	3 (8.1)	1.79	0.51–6.27	0.36
	1–4	837	68 (8.1)	1.61	1.07–2.44	0.02
	5–9	1633	160 (9.8)	1.94	1.35–2.8	<0.01
	10–14	1714	162 (9.5)	1.85	1.28–2.67	<0.01
	15–29	2080	121 (5.8)	1.19	0.82–1.72	0.37
^*^Sex	Female	3550	242(6.8)	1.00	—	
	Male	3553	311(8.8)	1.16	0.97–1.39	0.10
Onset week		7103		0.87	0.85–0.89	<0.01
LGA	Gusau	835	48(5.8)	1.00	—	
	Bakura	555	62(11.2)	1.91	1.28–2.84	<0.01
	Birnin Magaji	643	36(5.6)	0.52	0.32–0.83	0.01
	Bungudu	264	9(3.4)	0.48	0.23–0.99	0.05
	Kaura Namoda	671	63(12.4)	1.62	1.10–2.37	0.01
	Maradun	544	26(4.8)	0.50	0.30–0.83	0.01
	Tsafe	167	29(17.4)	3.37	2.04–5.58	<0.01
	Others	3424	260(7.6)	1.25	0.91–1.73	0.17

^*^Sex was included in the logistic regression model as a potential confounder with females as the reference. The reference for LGAs in the model was Gusau, the state Zamfara state capital. The reference for age group was adults >30 years old.

**Figure 3:**
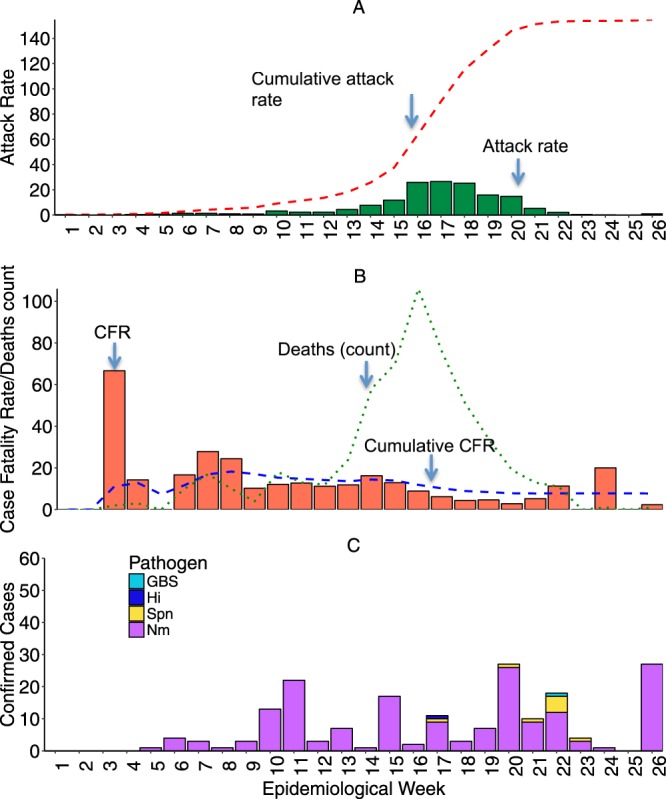
Epidemiologic curve of the meningitis outbreak in Zamfara State. The attack rates (**A**), case fatality rates (**B**) and laboratory-confirmed cases (**C**) by epidemiologic week. The distribution of pathogens detected among laboratory-confirmed cases is shown in (**C**).

The first suspected meningitis cases in Zamfara state occurred in December 2016 but were only reported at the national level in February 2017. By the 31^st^ of May 2017 the number of cases had increased to 7140. The highest attack rates exceeding 20 per 100,000 population were reported between epidemiologic weeks 16 and 18 (Fig. 3A). The bulk of the suspected meningitis cases 6,205 (86%) occurred between March and April 2017, which corresponds to the peak of hot dry harmattan winds.

Although fewer suspected cases were reported in the earlier weeks, the likelihood of death amongst suspected cases was highest then (Fig. 3B). Although the CFR declined to 10% or less after epidemiologic week 8, the absolute number of deaths increased substantially over the following weeks as the total number of suspected cases also increased. Half the deaths among suspected cases in Zamfara state occurred between epidemiologic weeks 9 and 12 (Fig. 3B). The odds of dying amongst suspected meningitis cases declined significantly over time (Table 2).

The first confirmed case was identified five weeks after the start of the outbreak, with meningococcus as the causing pathogen (Fig. 3C). All confirmed cases thereafter were attributed to meningococcus until the 17^th^ week of the outbreak when pneumococcus, GBS and *H. influenzae* were detected. Most pneumococcal meningitis cases, 7 (77%) were reported between weeks 20 and 23 (Fig. 3C).

### Demographic characteristics of the outbreak

**Figure 4:**
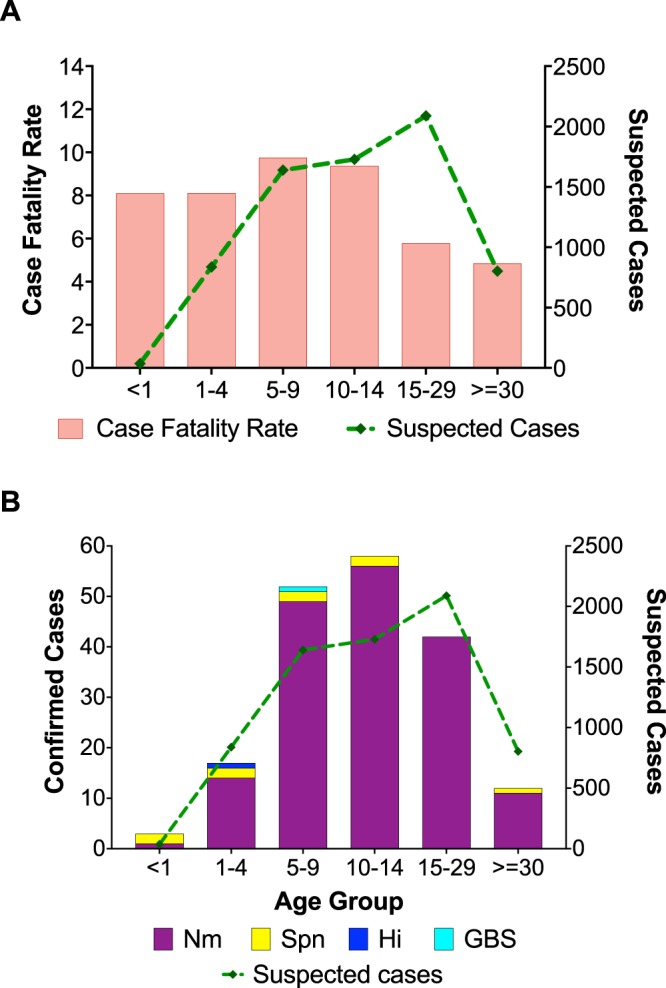
Case fatality rates and laboratory-confirmed cases by age group in Zamfara State. The case fatality rates and total suspected cases are shown by age group (**A**). The distribution of pathogens among laboratory-confirmed cases by age-group (**B**). The suspected case counts by age group are presented in Table 1.

Half of the suspected cases and 56% of the confirmed cases were males (Table 1). Male suspected cases died more frequently with a CFR of 8.7 compared to 6.8 among females. Males accounted for 311 (56%) of all deaths. Older children (5–14 years) and young adults (15–29 years old) accounted for 5,458 suspected cases, more than three-quarters of all suspected cases (Table 1). Older children between 5 and 14 years accounted for two-thirds (322/553) of the deaths reported. Likewise, the highest CFRs of 9.7 and 9.3 were reported among children 5–9 and 10–14 years old, respectively (Fig. 4A). Adults (≥30 years old) had the lowest CFR (4%). Suspected meningitis cases between the ages of 5 and 14 years old were nearly twice as likely to die than adults older than 30 years old (P < 0.01) (Table 2). Two of the three confirmed meningitis cases among infants were attributed to pneumococcus. There was at least one case of pneumococcal meningitis in all the age groups, except among the 15–29 year olds (Fig. 4B). The only cases of *H. influenzae* and GBS were reported in children aged 4 and 6 years. Two-thirds, of the confirmed meningococcus confirmed cases were among children between 5 and 14 years old (Fig. 4B).

## Discussion

We report the largest NmC meningitis outbreak to hit a single state in Nigeria to date. Zamfara state in North-western Nigeria accounted for more than half of the 14,280 suspected meningitis cases reported across the country during the reported period of the outbreak^20^. This outbreak followed three previous outbreaks that occurred in North-western Nigeria after the successful MenAfriVac mass vaccination campaigns. Prior to the recent outbreaks, the last NmC outbreak in sub-Saharan Africa was reported in 1979 in Burkina Faso^10^. The emergence and expansion of NmC ST-10217 CC with the propensity to cause large outbreaks threatens the public health gains made by the MenAfriVac campaigns^7,16^. This reinforces the need for strategic vision and action towards controlling meningitis epidemics in the meningitis belt.

Funk and colleagues reported NmC meningitis outbreaks that occurred sequentially in Sokoto (2013) and Kebbi (2014). These outbreaks were relatively small, with less than a thousand suspected cases and localised to specific regions in the affected states^12^. In 2015, larger outbreaks with 6,394 suspected cases were reported in Sokoto, Kebbi and Niger states^11^. Sidikou and colleagues also reported a large meningococcal meningitis outbreak with almost ten thousand suspected cases and 549 deaths in 2015^13^. The 2016–2017 outbreak in Nigeria was much larger with 553 deaths in Zamfara state alone. All previous outbreaks were attributed to a novel strain of NmC ST-10217 CC. As with previous outbreaks, sequenced NmC also belonged to the ST-10217 CC. In 2016, NmC meningitis localised outbreaks were reported in Niger and Nigeria along with sporadic cases reported in Cote d’Ivore, Burkina Faso and Ghana^21^. Brynildsrud and colleagues performed whole-genome sequencing on 150 meningococcal isolates collected during outbreaks in Nigeria and Niger between 2013 and 2017^16^. Their analysis which included four NmC isolates collected from meningitis cases from Zamfara State in 2017 confirmed the dominance of ST-10217. Brynildsrud and colleagues demonstrated that this hypervirulent NmC ST-10217 emerged through the acquisition of virulence factors and capsule genes^16^.

At a meeting on meningitis outbreak preparedness convened by WHO in 2015, global experts predicted that NmC outbreaks would continue to occur across the meningitis belt^17^. It may be possible that the current increase in NmC outbreaks is due to serogroup replacement following the mass MenAfriVac campaigns and this warrants further investigation. A key driver of this expansion is poor population immunity as carriage rates of NmC have historically been very low^22^. Furthermore, since the last NmC outbreak that occurred in the 20^th^ Century, there have only been a few mass vaccination campaigns targeted at NmC^17^. This is further compounded by the emergence of a novel virulent strain of NmC genetically distinct from previously described strains^11,14^. In light of this, the expansion in magnitude and spatial spread of NmC culminating in a large countrywide outbreak in Nigeria was predictable^23^.

There are several polysaccharide and conjugate vaccine formulations that include NmC. However, these vaccines have limited availability and are prohibitively expensive even at subsidized cost. The WHO currently recommends that the limited vaccine stocks be reserved for epidemics^24^. However, reactive vaccination is hugely dependent on strong and effective in-country surveillance systems with the capacity to rapidly and accurately detect, report and respond to meningitis outbreaks^13^. The revised WHO alert threshold is 3 confirmed cases per 100,000 per week for meningitis^24^. Unfortunately, many countries in the meningitis belt, including Nigeria, have weak primary health care and national disease surveillance systems. The first cases of meningitis in Zamfara state occurred in December 2016 but only reported to the national level in February 2017. The NmC vaccination campaigns did not start until April of 2017 at which point the outbreak had gone over its peak (Fig. 3).

One major constraint that delayed adequate and timely responses to the explosive meningitis outbreak was the lack of physicians to diagnose suspected meningitis cases and perform lumbar punctures. A small proportion, 6% of the suspected meningitis cases had lumber puncture performed (Fig. 1). Another important challenge was the poor logistical support for specimen transport. In the few LGAs where the doctors were skilled enough to perform LPs, there were no systems in place for specimen transport to the microbiology laboratories. As a consequence, culture was performed on only a third of CSF specimens collected and the culture positivity was also low at 9%. The requisite TI transport media was not always available and most laboratories in this state did not have the capacity to detect and characterize bacterial agents of meningitis. Strengthening of national disease surveillance systems and laboratories is key in minimizing vaccine-preventable deaths due to meningococcal meningitis in the future.

In previous NmC outbreaks in northern Nigeria and Niger, the highest proportions of cases (39–48%) were among children 5 to 14 years old^11–13^. Likewise, in this outbreak, children 5 to 14 years old accounted for nearly half of suspected meningitis cases and for an even higher proportion (60%) of deaths. The CFR among children 5 to 9 years old was 9.7, more than double the CFR among adults 30 years and older. These findings suggest that older children (5–14 years) may not only be more vulnerable to NmC meningitis but also more likely to die from it. WHO recommends vaccination of individuals 2–29 years during meningitis outbreaks^17^. However, targeted preventative mass vaccination campaigns for children 5–14 years in the most vulnerable communities may curtail NmC outbreaks and markedly reduce deaths.

An epidemiological curve similar to the 2015 Niger NmC outbreak^13^ was observed. The number of new suspected meningitis cases remained relatively stable until the 15th week after which there was a sharp rise in new suspected cases. In contrast, outbreaks caused by NmA, NmW and NmX tend to increase steadily peaking earlier in the epidemic season^19^. Slow initial progression followed by rapid acceleration to the peak of the outbreak, towards the end of the epidemic season may be a unique feature of NmC outbreaks. This pattern reinforces the need to have in place adequate outbreak preparedness plans alongside stockpiling medical and laboratory supplies including NmC-containing vaccines.

All five serotyped pneumococci belonged to serotypes included in the 10-valent pneumococcal conjugate vaccine (PCV10) implemented in Nigeria. Large pneumococcal outbreaks have been reported in Burkina Faso, Togo and northern Ghana in the past^25–28^ and more recently in the Brong Ahafo Region of Ghana^29^. West African pneumococcal outbreaks have characteristically high case-fatality rates (up to 40%) and appear to mimic meningococcal meningitis outbreaks. Although pneumococcus accounted for only 5% of confirmed cases in this outbreak, this finding highlights the need for surveillance and monitoring pneumococcal invasive disease in Nigeria.

An important limitation of this study is that only a very small proportion (6%) of the 7140 suspected cases had lumbar puncture performed and an even smaller fraction (5%) were tested in the laboratory. In addition, highly sensitive quantitative PCR testing was not performed on nearly half of the CSF specimens collected (Fig. 1). As a consequence, we probably missed a significant number of positive specimens. The meningococcal isolates were lost due to poor storage conditions in Zamfara state, therefore more extensive antibiotic resistance and genomic characterisation of the strains was not performed. Genotyping by MLST of the strains had to be performed directly from CSF specimens and this could only be done for two specimens due to limited funds. This report would have been enriched by the inclusion of more detailed clinical and vaccination history data of the suspected meningitis cases. For instance, we could not assess the clinical features such as meningococcaemia that could be associated with high mortality. Unfortunately, clinical and demographic data were not recorded routinely among suspected meningitis cases.

The NmC outbreak in Zamfara with over 500 hundred deaths is strong evidence of the on-going expansion of NmC epidemics. To curtail the spread of NmC outbreaks, governments and partner agencies should consider investing in targeted vaccination of the most vulnerable children (5–14 years old) and communities. However, sustainable control and elimination of meningitis outbreaks in the Africa meningitis belt may depend on the extension of the highly successful preventive MenAfriVac campaign framework to include multivalent conjugate meningococcal vaccine that covers the serogroups with the potential to cause outbreaks^7,30^. Licensed NmC conjugate vaccines remain largely unaffordable to countries facing the biggest risk of outbreaks. A polyvalent vaccine containing serogroups C and X conjugates has been developed by the Serum Institute of India and is currently undergoing clinical trials in Mali^31^. As the threat of large meningitis outbreaks looms with every harmattan season, a global action plan that will mobilise resources is urgently needed to strengthen surveillance and preparedness systems in Africa. Without such an action plan, it is likely that many more lives will be lost.

## Materials and Methods

### Study Area

Zamfara State is located in North-western Nigeria and has an estimated population of 3.85 million. The largest referral hospital, Ahmad Sani Yariman Bakura Specialist Hospital Gusau is in the state capital, Gusau. The state is divided into fourteen LGA. All LGAs, between December 2016 and May 2017, reported suspected meningitis cases. Zamfara has a Sahel Climate, mostly hot and dry, with temperatures rising as high as 40 °C between March and May. The Harmattan, hot and dry winds, lasts from February until the onset of the rains in June.

### Case definitions

Suspected meningitis was defined as sudden onset of fever (>38 °C) in combination with stiff neck, altered consciousness and/or petechial rash among patients older than one year. Among infants, suspected meningitis was defined as sudden onset of fever with bulging fontanel and/or petechial rash. Cerebrospinal fluid (CSF) was collected from suspected meningitis patients. Patients with suspected meningitis were treated with ceftriaxone as recommended by WHO^17^. Cerebrospinal fluid (CSF) was collected from patients meeting the case definition. Suspected cases were reported to the LGA and the State Disease Surveillance Team (DST).

### Bacteriologic analysis of CSF specimens

For bacteriologic analysis, 0.5–1.0 ml of patients’ CSF specimen were inoculated upon collection into each of Trans-Isolate (TI) medium when available, a plain tube and a calcium oxalate bottle and transported to the Ahmed Sani Yariman Bakura Specialist Hospital, Gusau within 24 hours. Upon arrival in the laboratory, an aliquot of the CSF specimen was centrifuged at 1,000 × g for 10 minutes and the supernatant was boiled for three minutes at 100 °C, cooled and used for Pastorex according to manufacturer’s instructions. The sediment was inoculated onto a plate 5% sheep blood agar and chocolate agar and used to prepare a smear for gram stain. White blood cell (WBC) count and CSF protein testing were done from the plain tube, and CSF glucose tested using the calcium oxalate samples. The improved Neubauer counting chamber was used for the WBC counts.

Following overnight incubation at 37 °C and in 5% CO_2,_ media plates were examined for growth characteristic of pneumococcus, *Haemophilus influenzae* and meningococcus. Alpha-haemolytic colonies were subjected to optochin (5 μg) testing; and smooth, glistening and moist colonies positive for the oxidase test subjected to Analytical Profile Index (API; BioMérieux SA, France) for confirmation as pneumococcus and meningococcus, respectively.

Serotyping of pneumococcal isolates was done using the latex agglutination method developed at MRCG as described previously^32^. Meningococcal serogroups were confirmed by slide latex agglutination serogrouping using Pastorex (Bio-Rad, UK) according to manufacturer’s protocol^33^.

### Molecular detection and serotyping

Molecular detection of pneumococcus, *H. influenzae* and meningococcus in CSF specimens was performed at the NCDC Laboratory, Abuja, Nigeria as previously described^29^. All CSF specimens analysed were also subjected to an *RNaseP* gene assay to confirm they were of human origin and to ascertain the integrity of the nucleic acids in the specimens. Specimens positive for *H. influenzae* and meningococcus were serotyped/serogrouped as previously described^29^.

CSF specimens positive for pneumococcus were shipped to the WHO CC at MRCG for molecular pneumococcal serotyping using the African scheme as described previously^34^. CSF specimens with CT values >32 were further subjected to conventional multiplex PCRs also described elsewhere^35,36^.

### Meningococcal Genotyping

MLST PCRs were carried out directly on only two of the CSF specimens that tested positive for NmC as previously described^37,38^. More specimens could not be tested due to limited funds. Nucleic acids were sent to Macrogen, Amsterdam Netherlands for amplification and sequencing of the seven housekeeping genes (*abcZ, adk, aroE, fumC, gdh, pdhC* and *pgm*).

### Data collection and analysis

Data collection was carried out using a customized version of the WHO immediate case-based report Integrated Disease Surveillance Report (IDSR) form and the Invasive Bacterial Vaccine Preventable Disease (IB-VPD) surveillance excel line list template. Demographic and clinical data were entered into the Microsoft Excel WHO approved IB-VPD surveillance line list template at the first reporting health facility. The completed ward line lists were collated and verified by the LGA by Disease Surveillance Notification Officers (DSNO) who in turn reported to the State Disease Surveillance Office. All CSF specimens were accompanied with a completed case-based report form in which laboratory results were appended. The Excel data sets from all LGAs were imported and merged into a Microsoft Access database. Pathogen detection and serotyping data were imported and merged with demographic and clinical data in the customised Microsoft Access database. Geographical Position System (GPS) coordinates of the Zamfara government levels (federal, state and LGAs) areas were downloaded from the United States of America Geographical Survey ((USGS), https://www.usgs.gov). Data analysis was carried out using R statistics tool (version 3.3.1) and Microsoft Excel. The maps were generated using QGIS v2.18.

A logistic regression model was applied to estimate odds ratios and their 95% confidence intervals for the associations between mortality and potential risk factors including age, timing (week) of disease onset and LGA of suspected cases. Sex was used as predefined potential confounder in the model. All analyses were carried out using Stata/SE 14.1 (Stata Corp. Texas, USA). P-values < 0.05 have been taken to indicate statistical significance.

### Ethics approval and consent to participate

All cases of suspected meningitis were defined following WHO protocol. The study was approved by the scientific coordinating committee of MRC Unit The Gambia and the joint MRC Unit/Gambian Government Ethics Committee (reference number SCC1188). All surveillance participants including parents/guardians of all children gave informed consent prior to enrolment.

The MRC Unit The Gambia, molecular microbiology laboratory submits to the external quality assurance programme of the UK National External Quality Assessment Service (http://www.ukneqas.org.uk) and is a World Health Organization (WHO) Regional Reference Laboratory for invasive bacterial pathogens.

All the methods in this study, including clinical and laboratory were carried out using WHO protocols.
